# Effects of harvesting on spatial and temporal diversity of carbon stocks in a boreal forest landscape

**DOI:** 10.1002/ece3.751

**Published:** 2013-09-09

**Authors:** Michael T Ter-Mikaelian, Stephen J Colombo, Jiaxin Chen

**Affiliations:** Ontario Forest Research Institute, Ministry of Natural Resources1235 Queen Street East, Sault Ste. Marie, Ontario, P6A 2E5, Canada

**Keywords:** Forest age, forest carbon, forest harvesting, managed forest, wood products

## Abstract

Carbon stocks in managed forests of Ontario, Canada, and in harvested wood products originated from these forests were estimated for 2010–2100. Simulations included four future forest harvesting scenarios based on historical harvesting levels (low, average, high, and maximum available) and a no-harvest scenario. In four harvesting scenarios, forest carbon stocks in Ontario's managed forest were estimated to range from 6202 to 6227 Mt C (millions of tons of carbon) in 2010, and from 6121 to 6428 Mt C by 2100. Inclusion of carbon stored in harvested wood products in use and in landfills changed the projected range in 2100 to 6710–6742 Mt C. For the no-harvest scenario, forest carbon stocks were projected to change from 6246 Mt C in 2010 to 6680 Mt C in 2100. Spatial variation in projected forest carbon stocks was strongly related to changes in forest age (*r* = 0.603), but had weak correlation with harvesting rates. For all managed forests in Ontario combined, projected carbon stocks in combined forest and harvested wood products converged to within 2% difference by 2100. The results suggest that harvesting in the boreal forest, if applied within limits of sustainable forest management, will eventually have a relatively small effect on long-term combined forest and wood products carbon stocks. However, there was a large time lag to approach carbon equality, with more than 90 years with a net reduction in stored carbon in harvested forests plus wood products compared to nonharvested boreal forest which also has low rates of natural disturbance. The eventual near equivalency of carbon stocks in nonharvested forest and forest that is harvested and protected from natural disturbance reflects both the accumulation of carbon in harvested wood products and the relatively young age at which boreal forest stands undergo natural succession in the absence of disturbance.

## Introduction

Rapid climate change resulting from increasing concentrations of greenhouse gases in the atmosphere and possible measures to curb emissions of carbon-based gases such as carbon dioxide and methane are perhaps the most discussed topics in the current literature. Forests, in addition to their ecological functions and multiple benefits to society, store large amounts of carbon and exert strong control over the global carbon cycle (Pan et al. 2011). Therefore, assessing forest carbon stocks and projecting how they will change are needed to develop forest management strategies that can contribute to mitigating climate change.

Ontario's forests cover over 71 million ha (Watkins 2011), most of them classified as boreal (50 million ha) or transition (20 million ha) forest. The latter, which lies between the boreal and deciduous biomes, is referred to as the Great Lakes-St. Lawrence forest. The remaining 1 million ha is deciduous forest in the south. In total, about 40.5 million ha of Ontario's forest receive some form of management (Ontario Ministry of Natural Resources [OMNR] 2012). Their vast extent, representing 2% of the world's forests (Watkins 2011), makes them very significant contributors to the global carbon cycle. In addition to storing carbon, sustainably managed forests can indirectly help reduce atmospheric greenhouse gases by storing carbon in wood products. In a study by Chen et al. (2010), the combined carbon stocks in Ontario's managed forests and harvested wood products were estimated to increase by 465 Mt C (millions of tons of carbon) by the end of the 21st century, with the increase mostly attributed to carbon storage in harvested wood products in use and in landfills and the regrowth of harvested forests.

The study by Chen et al. (2010) was based on projections of future forest harvesting levels and changes in forest age structure and species composition contained in forest management plans from circa 2000. In that analysis, projected harvesting levels were modified from those contained in forest management plans: The managed forest was divided into two regions and harvested area was adjusted using a region-wide factor reflecting Ontario's recent average harvesting levels. Based on these adjusted harvesting projections, Chen et al. (2010) produced a trajectory of future carbon stocks in forests and harvested wood products.

Actual harvesting levels fluctuate considerably both spatially and temporally across Ontario, reflecting differences in industrial demand for wood of a given tree species. Meanwhile, harvesting level has been demonstrated to have a major effect on landscape-level forest carbon dynamics (e.g., Bradford 2011; Krankina et al. 2012). The objective of this study was to (1) estimate existing forest carbon stocks across a globally significant managed North American boreal forest landscape, and (2) examine the effects of various harvesting levels on projected dynamics of carbon stocks in forests managed for production of fiber. We projected a range of feasible trajectories of future forest carbon stocks for this landscape based on five scenarios of forest harvesting, reflecting variation in historical harvesting levels and their spatial differences. Finally, we evaluated potential wood product carbon stocks and how their inclusion affects total forest carbon stock estimates (forest ecosystem carbon plus wood product carbon).

## Materials and Methods

**Figure 1:**
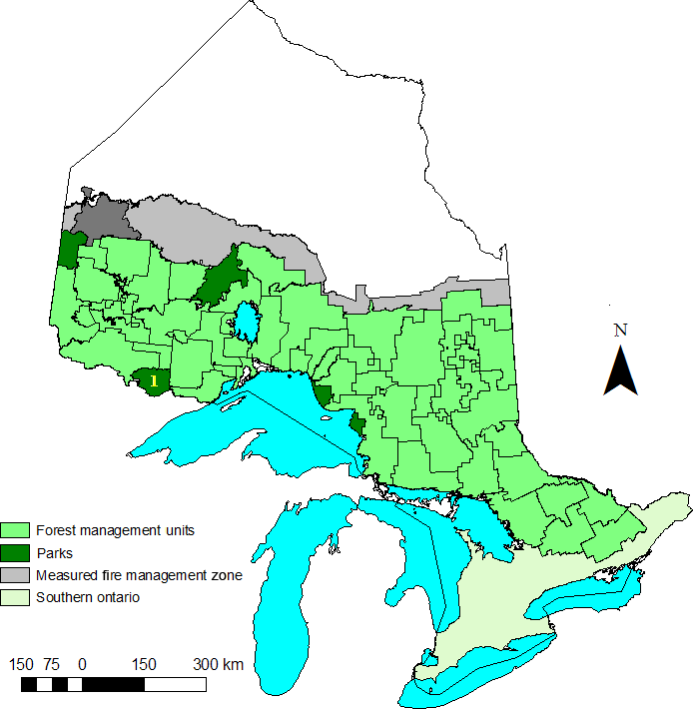
Major categories of forested land in Ontario: Crown forest where harvest is allowed (green, with black lines boundaries between forest management units); large parks (dark green), measured forest management zone (gray), and private forest in southern Ontario (light green). Dark gray area, Whitefeather; 1, Quetico Provincial Park.

In this study, we examined current and projected carbon stocks in managed forests in Ontario, Canada. In determining what to include in managed forests we used the Intergovernmental Panel on Climate Change definition (The Intergovernmental Panel on Climate Change [IPCC] 2003), in which “forest management is the process of planning and implementing practices for stewardship and use of the forest aimed at fulfilling relevant ecological, economic and social functions of the forest.” Thus, managed forests were those receiving any one of the full spectrum of management, from commercial timber production to stewardship for noncommercial purposes. Based on level of management received, Ontario's forests were categorized as: (1) crown, that is, state-owned forest where commercial harvesting is allowed, but where much of the forest is also protected for nontimber values (Colombo et al. 2012); (2) large parks, where harvesting is not permitted and fire may be suppressed if it threatens timber or infrastructure outside the park; (3) measured fire management zone, where commercial harvesting is not permitted and fires are often left to burn; and (4) privately owned forests (Fig. 1). Estimation of forest carbon stocks for each of these categories is described in detail below.

### Crown forest where harvesting is allowed

For management purposes, Crown forest that can be harvested is divided into 43 (as of 2010) forest management units (FMU) covering a total forest area of 28.3 million ha. Within these managed forest areas over 4.7 million ha, or 16.5% of the total forest area, is protected from harvesting (e.g., small parks and other reserves).

Individual FMUs range from about 188,000 to 2,660,000 ha and from hardwood-dominated stands in the Great Lakes-St. Lawrence forest region to conifer-dominated boreal forests further north. These FMUs are managed by individual forest companies following regulations set by Ontario's Ministry of Natural Resources. In these forests, harvested areas are allowed to regenerate naturally resulting in a diversity of species and therefore stands that differ from the simplified structure associated with intensive forestry elsewhere in the world.

Crown forests available for harvesting were described using Ontario's Forest Resource Inventory, an aerial survey providing information about the extent, species composition, and age-class structure of the forest (Gillis and Leckie 1993). In the inventory, individual forest stands of similar species composition that developed in a similar manner and are managed under the same silvicultural system are aggregated into forest units (Ontario Ministry of Natural Resources [OMNR] 1996). These forest units are further aggregated into 10-year age classes. This processed forest resource inventory is used as an input into a planning model, the Strategic Forest Management Model (SFMM) (Kloss 2002). This model is used to simulate forest development through time for each forest unit by assigning natural disturbance rates based on regional disturbance history, succession rules defining transition of stands from one forest type and/or age class to another, and yield curves (Plonski 1981; Penner et al. 2008). In addition, the model is set up to account for detailed silvicultural rules and prescriptions (e.g., harvesting eligibility based on stand age or volume, renewal methods, tending and rehabilitation treatments), management objectives (e.g., wood supply, financial resources), and environmental constraints (e.g., diversity of future forest, desired future forest condition, potential for wildlife habitat). The simulation that maximizes timber volume available for harvesting while meeting environmental constraints is deemed the preferred management strategy. This strategy forms the basis for the forest management plan in the FMU. Hereafter, this simulation is referred to as the maximum harvesting scenario (MH).

**Figure 2:**
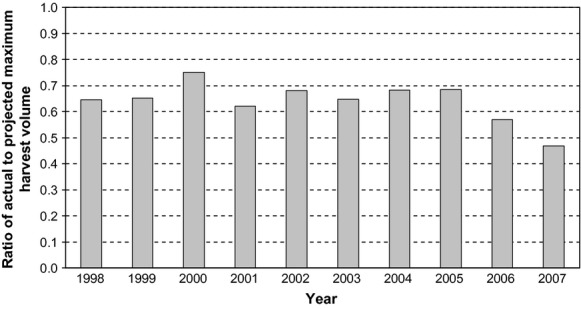
Ratio of actual to projected maximum harvest volume for all forest management units in Ontario based on pooled 1998–2007 data for softwood and hardwood.

In Ontario, forest management plans are updated on a 5-year cycle, incorporating the most recent data from actual harvesting levels and area affected by natural disturbances during the preceding 5 years. In this study we used the most recent forest management plans, ranging in year of development among FMUs from 2004 to 2010; plans dated 2004 and 2005 were used for only 1 and 4 FMUs, respectively, and the remainder were from 2006 to 2010. MH scenarios from management plans predict the greatest forest area and wood volume that can be harvested while meeting targets for other aspects of sustainability of forest ecosystems. However, in practice maximum volumes are rarely harvested due to factors such as low market demand and cost of harvesting/lack of access to stands in remote areas. Analysis of ratios of actual harvested volumes to those specified in MH scenarios for 1998–2007 (J. Maure, pers. comm.) showed considerable variation over time, both among FMUs and between softwoods and hardwoods. Figure 2 shows the ratios of actual to projected MH volumes for all FMUs combined. Among individual FMUs these ratios varied from 0.308 to 1.0 for softwoods and from 0.200 to 1.0 for hardwoods. The upper limit (1.0) occurred in only one FMU. The 10-year mean ratio of actual to projected MH volume averaged over all FMUs and softwoods and hardwoods was 0.640.

Using data for 1998–2007, we estimated historical average (HA), historical low (HL), and historical high (HH) harvesting ratios separately for softwood and hardwood for each individual FMU. HL and HH harvesting ratios were estimated as the minimum and maximum annual ratio of actual to MH volumes, respectively, observed between 1998 and 2007. For the same period, the HA ratio was estimated as the sum of total actual harvested volume to the maximum total harvested volume. Estimated ratios were used to set new target harvest volumes for each FMU and to rerun SFMM to produce HA, HL, and HH scenarios of forest growth and harvesting. We also used a no-harvest scenario (NH) in which harvest level was set at zero and forest dynamics was affected only by natural disturbance. Parameters describing forest growth (succession rules, yield curves, and disturbance rate) were kept the same in all five simulated scenarios.

The forest simulations resulting from these harvesting scenarios were used to predict forest carbon stocks in Crown forest where harvesting is allowed. Some constraints present in the MH scenarios used in forest management planning were relaxed in the HL, HA, and HH scenarios to let SFMM simulations converge to optimum solutions. Thus, despite retaining most elements required in forest management plans, the HL, HA, and HH scenarios used here should not be construed as modified forest management plans.

### Large parks

Seven large parks, ranging in area from 18,900 ha (Michipicoten Island) to 890,100 ha (Wabakimi Provincial Park), are not eligible for harvesting. (Harvesting is allowed in an 8th large park, Algonquin Provincial Park, which is treated as an FMU.) Of the seven large parks, forest resource inventory that provides sufficient data on forest species composition and age structure to estimate forest carbon stocks has been completed only for Quetico Provincial Park. For the other large parks, the amount of forested area was estimated using a land-cover database (Watkins 2011). Unfortunately, data on forest age structure and species composition in the land-cover database are insufficient to directly estimate forest carbon stocks. Therefore, with the exception of Quetico Provincial Park, the estimate of forest carbon stocks for large parks was extrapolated using carbon stock density estimated for other protected areas in FMUs in the ecoregion in which a given park is located.

### Measured fire management zone

The measured fire measurement zone borders the northern edge of the FMUs (Fig. 1). Unlike FMUs and large parks, in which fire is aggressively suppressed to minimize area burned, in the measured fire management zone the decision to suppress fire is based on the values at risk, the fire's growth potential, and the expected cost. In this zone, if initial suppression efforts fail, an assessment of these factors may result in a decision to let the fire burn (Ward et al. 2001).

The total area of the measured fire management zone is 7,654,300 ha, of which 5,268,900 ha are forested. Forest inventory has been completed only in an area referred to as the Whitefeather Forest (Fig. 1), which covers 958,600 ha, allowing direct estimation of forest carbon stocks for this area. This information was used to provide a carbon density estimate that was applied to estimate carbon stocks for the remainder of the measured fire management zone.

### Private forest

Private forest occupies 4,136,400 ha within the FMUs. Forest resource inventory is available for all private lands within the FMUs, allowing forest carbon stocks for this area to be estimated. However, forest resource inventory is not available for private forest lands in southern Ontario (south of the FMUs and approximately south of 45°N). Forested area in southern Ontario covers ∼1.2 million ha and has been classified into land-cover types based on satellite imagery (Watkins 2011). Similar to the approach used for large parks for which forest resource inventory was unavailable, current forest carbon stocks in privately owned forests in southern Ontario were estimated using average forest carbon density of the three FMUs bordering the southern Ontario private forests.

### Estimating forest carbon stocks

Forest carbon stocks were estimated using the large-scale forest carbon budget model, FORCARB-ON, that was developed to estimate current and future carbon stocks in forests and harvested wood products (Chen et al. 2010). The model can be used to estimate forest carbon stocks in six pools: live and standing dead trees (above- and belowground), down wood (i.e., large [>76 mm diameter] logs and branches and stumps), understory vegetation, forest floor (dead organic matter above the mineral soil horizon, including small [<76 mm diameter] branches and logs, litter, and humus), and soil. The main predictors of carbon densities in various pools are merchantable volume or forest age by species group. Estimates of carbon stored in live and standing dead trees are based on net merchantable volume. Estimates of carbon in down wood are based on relationships with live tree biomass that rely on factors such as mortality rates of live trees, ratios of down wood carbon to live tree carbon, and down wood decay factors. Forest floor and understory vegetation carbon pools are estimated based on stand age. Finally, soil carbon includes all organic carbon (excluding roots) in mineral soil to 1 m depth and is estimated using forest area and soil carbon density based on forest region and forest type. For additional detail on FORCARB-ON estimates of forest carbon pools, see Chen et al. (2010).

FORCARB-ON can be used in two modes (1) using SFMM simulations and (2) forest resource inventory as model input. For areas for which SFMM simulations are available, SFMM data and results are used as inputs (first mode). Data on area distribution by forest unit and age and projected harvest volumes are used to estimate current and predict future forest carbon stocks. This approach was applied in this study for individual FMUs. For areas not included in SFMM projections but for which forest resource inventory was available (i.e., Quetico Provincial Park, Whitefeather Forest, private land in FMUs), algorithms and parameters used in FORCARB-ON were used to calculate carbon yield curves that predicted total amount of carbon (e.g., sum of six carbon pools simulated by FORCARB-ON) from yield curves assigned to stands in the forest resource inventory (second mode). These carbon yield curves were used along with forest inventory to estimate current forest carbon stocks in inventoried areas. Finally, present forest carbon stocks for noninventoried areas (large parks other than Quetico Provincial Park and the measured fire measurement zone outside of Whitefeather) were estimated by applying estimated present forest carbon density (total forest carbon stock per ha) for protected areas in the ecoregion in which each large park was located and for Whitefeather, respectively. No attempt was made to predict future dynamics of forest carbon stocks in the measured fire management zone, other large parks, or private lands due to the lack of information about future growth and management of these forests.

FORCARB-ON was also used to estimate carbon stocks and emissions for harvested wood products in four end-use categories: (1) products in use, (2) products and processing residues disposed of in landfills, (3) wood burned to generate energy, and (4) wood burned without producing energy and wood decomposition (Chen et al. 2008). Allocation of carbon in harvested wood products to the four end-use categories was estimated using harvest volumes projected by SFMM for Crown forest land where harvesting is allowed. A carbon distribution matrix in FORCARB-ON allocates harvested wood over the four categories and subsequently transfers carbon from in use to other categories and from landfill to emissions. We also estimated carbon stocks in historical harvested wood products using data for 1951–1990 from Chen et al. (2008) and annual harvested volumes in Ontario for 1991–2008 (J. Maure, pers. comm.).

Carbon stocks in forest and harvested wood products were estimated using the 10-year simulation step in SFMM. As the year of forest management plan development varied from 2004 to 2010 among FMUs, we used 2010 as a reference year by interpolating predicted carbon stocks for two consecutive output steps bracketing 2010. Prediction results are presented for the period 2010–2100. Similarly, forest resource inventory for areas not included in SFMM simulations (i.e., Quetico Provincial Park, Whitefeather Forest, private lands) was advanced, if needed, by increasing the age of each inventoried stand to what it would have been in 2010.

The effect of forest age and harvesting rate on carbon density change was tested for the HA scenario using the following variables calculated separately for each FMU. Change in forest age was calculated as the difference between area-weighted average age of forest in 2100 and 2010. The mean decadal harvest rate was calculated as the ratio of projected annual harvested volume within a 10-year term to the growing stock at the beginning of the term, averaged over all terms from 2010 to 2100. The total harvesting rate was calculated as the ratio of total harvested volume projected for 2010–2100 to the growing stock at 2010 divided by 90 (length of simulation period). Both harvesting rates have units (m^3^ m^−3^ year^−1^) and were expressed as percentage of growing stock.

## Results

**Figure 3:**
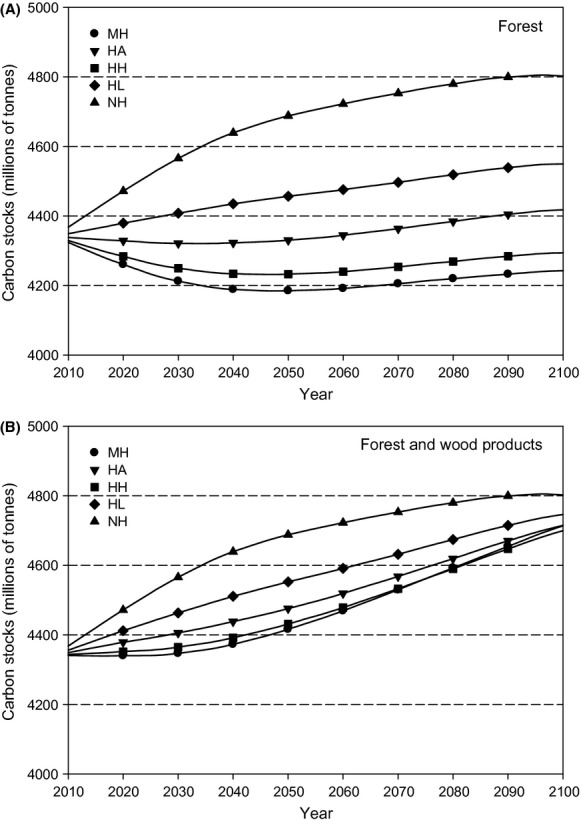
Carbon stocks in Ontario's forest where harvest is allowed (A) excluding and (B) including carbon stored in harvested wood products in use and in landfill for no-harvest (NH) and four harvesting scenarios: MH, maximum harvest; HA, historical average; HL, historical low; HH, historical high (as defined in text).

Total carbon stocks in Ontario's managed forests estimated for 2010–2100 for five simulated scenarios are presented in Table 1. Carbon stocks in Crown forest where harvesting is allowed and in harvested wood products (in use and in landfill) are shown in Figure 3. Altogether, managed forests and harvested wood products were projected to store about 6.4 billion tons of carbon in 2010. By far the largest portion of carbon in managed forests (68%) was stored in Crown forest where harvesting is permitted. A further 11% was stored in the measured fire management zone and 15% in private forest land. Small differences among scenarios in carbon stock estimates for 2010 occurred because simulated scenarios for forest where harvesting is allowed were based on management plans which usually started before 2010 (Fig. 3 and Table 1).

**Table 1:** Carbon stocks (million tons of C) in Ontario's managed forests and wood products for five simulation scenarios for 2010–2100

			Year										
				
Carbon stocks category	Area (million ha)	Scenario	2010	2020	2030	2040	2050	2060	2070	2080	2090	2100	Total change^1^
Crown land where harvest is allowed	28.28	NH	4367.9	4471.6	4565.5	4639.1	4687.7	4722.5	4752.7	4779.5	4799.2	4802.0	434.2
		HL	4349.0	4379.3	4407.8	4434.7	4456.6	4475.6	4496.4	4518.6	4538.8	4549.7	200.7
		HA	4338.2	4328.7	4321.3	4322.6	4330.3	4344.4	4363.3	4384.2	4404.4	4417.5	79.3
		HH	4329.2	4283.9	4249.6	4233.8	4232.8	4239.8	4253.5	4269.1	4284.0	4293.9	−35.3
		MH	4323.9	4260.6	4212.5	4188.7	4185.0	4191.5	4204.9	4219.7	4232.6	4242.6	−81.3
Large parks	1.67		261.3	261.3	261.3	261.3	261.3	261.3	261.3	261.3	261.3	261.3	0.0
Measured fire management zone	5.27		711.5	711.5	711.5	711.5	711.5	711.5	711.5	711.5	711.5	711.5	0.0
Private forest land in managed forest area	4.14		667.9	667.9	667.9	667.9	667.9	667.9	667.9	667.9	667.9	667.9	0.0
Private forest land in southern Ontario	1.20		237.3	237.3	237.3	237.3	237.3	237.3	237.3	237.3	237.3	237.3	0.0
Future wood products from managed Crown forest	N/A	HL	6.8	32.7	55.3	75.9	95.6	115.3	135.1	155.2	175.6	196.1	189.3
		HA	10.7	49.9	84.2	115.5	145.2	174.7	204.4	234.6	265.7	296.8	286.1
		HH	14.4	68.1	115.1	157.9	198.3	238.6	279.0	320.3	362.9	405.5	391.1
		MH	16.8	79.5	134.2	184.0	230.9	277.4	324.5	372.5	421.7	471.1	454.3
Historical wood products (1950–2010)	N/A		142.2	138.1	134.2	130.6	127.6	125.0	122.8	121.1	119.2	117.7	−24.5
Total carbon stock in managed forest and wood products	40.56	NH	6388.1	6487.8	6577.7	6647.8	6693.4	6725.5	6753.6	6778.6	6796.4	6797.7	409.6
		HL	6376.0	6428.1	6475.3	6519.3	6557.9	6593.9	6632.3	6672.8	6711.7	6741.5	365.4
		HA	6369.1	6394.8	6417.7	6446.8	6481.1	6522.0	6568.6	6617.9	6667.3	6710.0	340.9
		HH	6363.8	6368.1	6376.9	6400.3	6436.7	6481.3	6533.4	6588.5	6644.1	6695.1	331.3
		MH	6360.9	6356.2	6359.0	6381.3	6421.5	6471.9	6530.2	6591.2	6651.4	6709.4	348.5

1 Total change refers to the difference in carbon storage between 2010 and 2100.

By 2100, carbon stocks in forests where harvesting is allowed were expected to change depending on harvest level. Stocks would decrease to 4243 Mt C and 4294 Mt C for the higher harvesting level scenarios (MH and HH, respectively) (Table 1 and Fig. 3). In comparison, harvesting at the HA and HL levels would see stocks increase to 4418 Mt C and 4550 Mt C, respectively (Fig. 3A). For all harvesting scenarios, wood product carbon stocks increased over time, reaching values ranging from 196 Mt C to 471 Mt C in 2100 depending on scenario. When forest carbon and carbon stored in harvested wood products were combined, stocks increased for all four scenarios, with values in 2100 ranging from 4714 Mt C to 4746 Mt C (Table 1 and Fig. 3B).

Forest carbon density was strongly affected by forest management. For example, the lowest carbon density in 2010 was 135 tons of carbon per ha (t C ha^−1^) in the measured fire management zone, where large fires have been allowed to burn out of control (Table 1). In comparison, the managed forest where harvesting occurs but fires are suppressed had a carbon density averaging 153 t C ha^−1^. Management in large parks excludes both harvesting and fire, which produced a carbon density of 156 t C ha^−1^. The much higher carbon density (198 t C ha^−1^) of private forest in southern Ontario reflects the more carbon dense deciduous forest biome (Peng et al. 2000).

**Figure 4:**
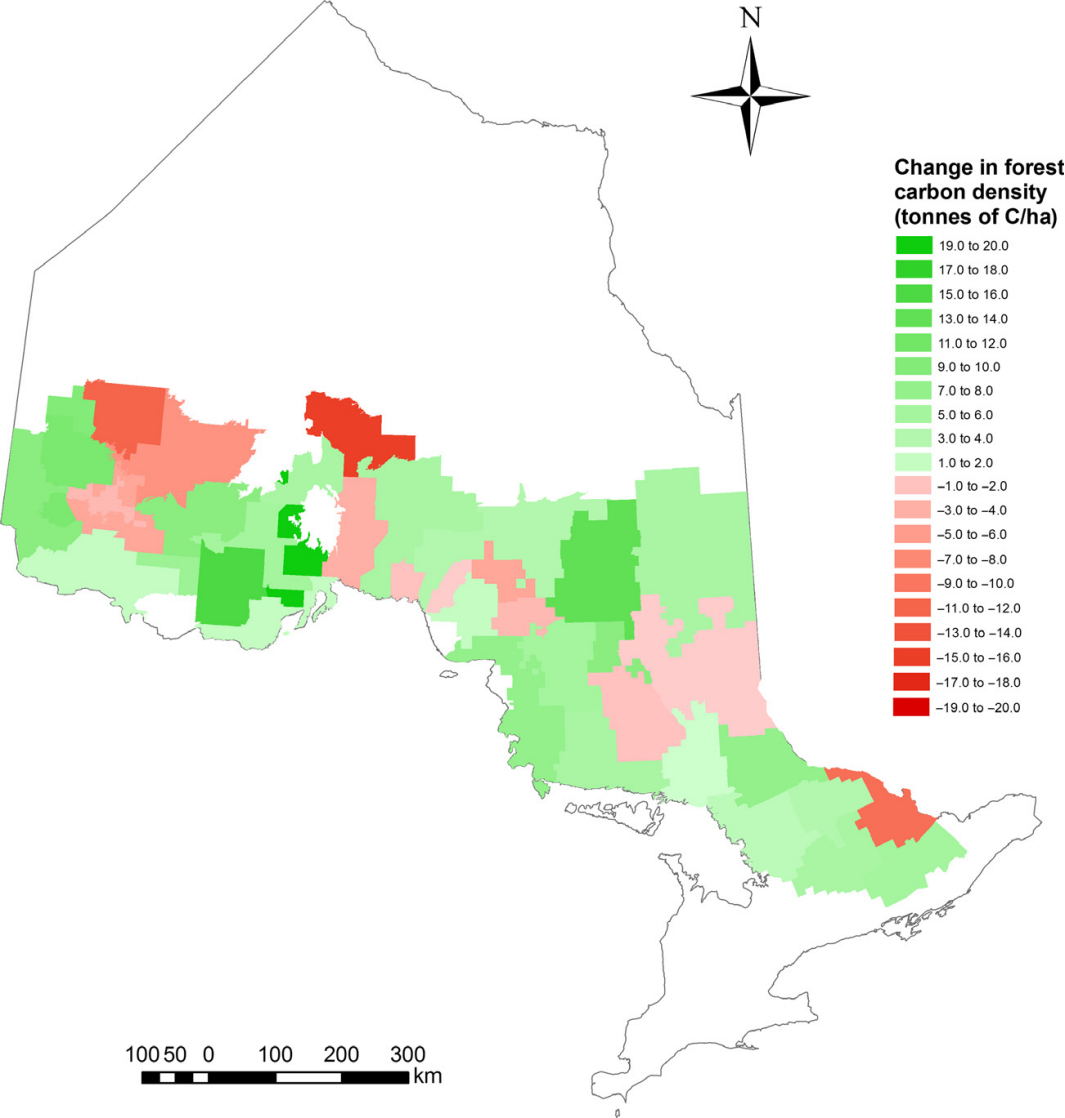
Projected changes in forest carbon density (tons of C per ha) from 2010 to 2100 in Ontario's Crown forest where harvest is allowed for historical average (HA) harvesting scenario. For each forest management unit (FMU), forest carbon density was calculated as total carbon stocks in Crown forest divided by total area, excluding private land forest within the FMU boundaries.

Not only did provincial average carbon density vary with harvesting level, the direction and degree of change in forest carbon density also varied considerably among FMUs. For the HA scenario, projected changes in forest carbon density ranged from −15.3 t C ha^−1^ to 19.3 t C ha^−1^ (Fig. 4). The number of FMUs for which carbon density was projected to increase was 16 compared to 27 where it was expected to decrease (Fig. 4).

**Figure 5:**
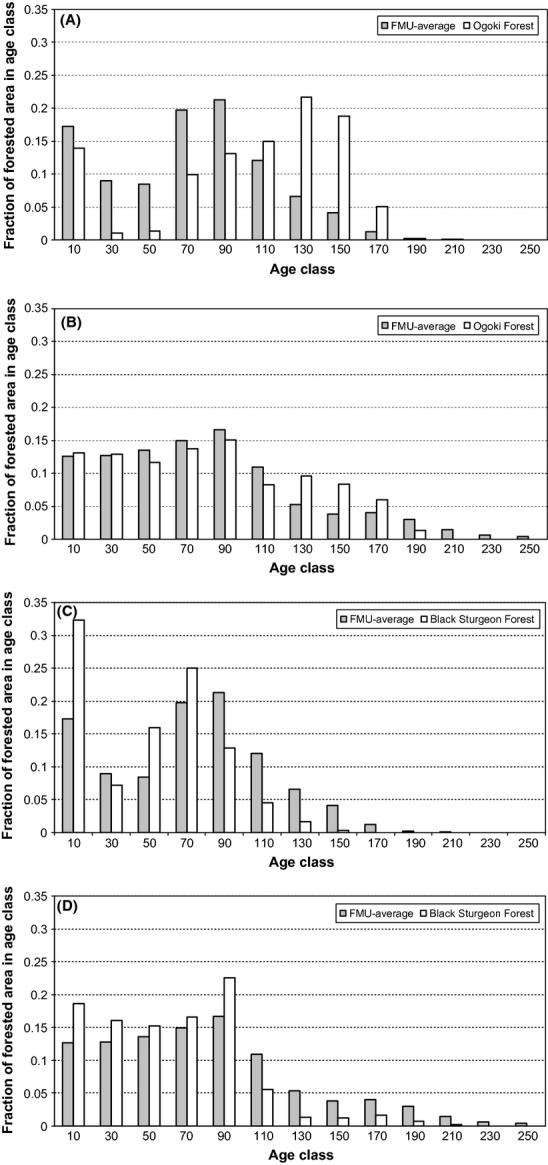
Forest age structure for two forest management units (FMU) (light bars) and average for all FMUs (gray bars) in Ontario, Canada. Age structure for: Ogoki Forest in 2010 (A) and 2100 (B); Black Sturgeon Forest in 2010 (C) and 2100 (D).

The mean decadal harvest rate and total harvesting rate varied among individual FMUs from the minima of 0.23% and 0.23% to maxima of 1.75% and 1.63%, with the mean values of 0.81% and 0.79%, respectively. Correlation between forest carbon density change from 2010 to 2100 and the mean decadal and total harvesting rates was equal to −0.045 and 0.111, respectively. Change in average forest age from 2010 to 2100 varied among individual FMUs from −22.4 years to 45.9 years (with the mean of 7.8 years) and were closely related (*r* = 0.603) to changes in forest carbon density over time (Fig. 4). Two FMUs – Ogoki Forest and Black Sturgeon Forest – projected to decrease (−15.3 t C ha^−1^) and increase (19.3 t C ha^−1^) their forest carbon density the most between 2010 and 2100, also had the oldest and youngest average forest ages in 2010 (103.0 and 48.9 years, respectively). Figure 5 shows forest age structure in the Ogoki and Black Sturgeon forests relative to average age structure for all FMUs in 2010 and 2100. In 2010, forest age structure in the Ogoki Forest was skewed toward older age classes relative to the average for all FMUs. In 2100, older age classes were still expected to be more prominent in the Ogoki Forest than across all other FMUs, but the difference was diminishing by 2010. The trend for the Black Sturgeon Forest was the reverse; younger age classes were more prominent in 2010 compared to the average FMU age structure, but again the difference mostly disappeared by 2100.

## Discussion

Estimated carbon stocks (Table 1) for four scenarios involving harvest are in the same range, albeit slightly lower, than those produced by Chen et al. (2010). For all managed forest, excluding private forest in southern Ontario, total carbon stocks in 2010 for the average harvesting level scenario were estimated at 5979 Mt C versus 6236 Mt C reported by Chen et al. (2010), and were projected to increase to 6058 Mt C and 6257 Mt C by 2100 in this study and by Chen et al. (2010), respectively. These differences reflect a combination of factors related to improved estimates of forested areas, updated forest inventories, and modified estimation methods. An earlier accounting of Ontario forest carbon stocks for 1990 (Liu et al. 2002) used a different model, different total forest area, and unique data sources for forest condition, growth, and disturbance, and thus produced results that were not comparable with those from this study or Chen et al. (2010), as discussed by Chen et al. (2010).

Estimated average carbon density for all FMUs was similar to values reported elsewhere. For example, average total carbon densities estimated by Stinson et al. (2011) were about 150 t C ha^−1^ for Boreal Shield West, 200 t C ha^−1^ for Boreal Shield East, and 220 t C ha^−1^ for the Mixedwood Plains in Canada. Average total carbon densities for similar areas in this study were 147.3 t C ha^−1^, 152.8 t C ha^−1^, and 197.3 t C ha^−1^, respectively. Estimated values were also within the range of values reported in the literature as summarized by Chen et al. (2010).

Projected carbon stocks in forest and harvested wood products were simulated as a range of values reflecting historical harvesting levels (Table 1). The difference in forest carbon stocks between simulated harvesting scenarios increased with time, reaching the value of 307 Mt C in 2100 between HL and MH scenarios. The difference in carbon stocks in harvested wood products is substantial as well (275 Mt C by year 2100 between HL and MH scenarios), with the sign opposite to that for forest carbon stocks: higher future harvesting rate results in lower forest carbon stocks, but higher stocks in harvested wood products. Although the range of values does not qualify as a statistically rigorous confidence interval, it nevertheless provides a measure of uncertainty resulting from one of the major factors affecting future carbon stocks in forests and harvested wood products. Values in Table 1 represent the same scenario for all individual FMUs (e.g., values for HL scenario in Table 1 are sums of values for HL scenarios for individual FMUs). This approach is more simplistic than using permutations of different scenarios for individual FMUs. However, it was based on the assumption that market forces dictating future harvesting rates stem from the provincial level rather than locally.

The effect of changes in forest age structure on carbon stock dynamics in this study matches findings by Böttcher et al. (2008), who modeled carbon stock changes in a hypothetical forest landscape. They demonstrated that, under the same management regime, the trend in carbon stocks depended on landscape starting age structure: a “young” landscape (with an age structure shifted toward younger age classes) accumulated carbon stocks, whereas in an “old” landscape (with an age structure shifted toward older age classes) carbon stocks declined. Similar results were produced by Hennigar et al. (2008) in a study with three forest age structures (young, even-aged, and old forests), who reported that landscape-level carbon sequestration rates decreased as the age structure shifted toward older stands. Vetter et al. (2005) studied age structure as an integrating effect of past management and attributed 8–17% of changes in biomass carbon balance in a large (about 0.5 million ha) study area in central Europe to forest age.

In Crown forest where harvesting is permitted, stand-replacing natural disturbance is projected to affect a very limited area, meaning that changes in forest age result primarily from harvesting and natural succession. Although harvesting level has an obvious effect on future dynamics of forest carbon stocks (e.g., compare results for five simulated scenarios in Table 1 and Fig. 3), contrary to our expectations, harvesting rate was poorly related to projected forest carbon density changes at an individual FMU level. A possible reason for this weak correlation is that “older” FMUs will undergo successional changes resulting in older stands being replaced naturally by younger ones. In the Ogoki and Black Sturgeon FMU examples described previously, total harvested area for 2010–2100 was comparable, affecting 40% and 50% of total forest area, respectively. Meanwhile, total area undergoing natural successional changes during the same period was 69% in the Ogoki versus 22% in the Black Sturgeon Forest. Thus, the “older” Ogoki Forest was projected to lose carbon due to successional changes, whether or not harvesting occurred.

The relative effect of forest age and harvesting rate on changes in forest carbon stocks is likely to depend on the length of period over which these changes are considered. The main harvesting method used in Ontario's Crown forest north of the transition forest is clearcutting, which reduces live tree carbon stocks to very low levels compared to natural succession where most of the mature tree canopy is generally retained. Therefore, the short-term effect of harvesting rate on forest carbon stocks is greater than that of succession-induced changes in forest age (Ter-Mikaelian et al. 2013). However, over longer periods, older forests are more affected by successional changes than younger ones, resulting in higher relative importance of forest age than harvesting rate.

Analyzing forest ecosystem carbon stocks without considering carbon stored in harvested wood products can lead to erroneous conclusions about the net effect of forest management on atmospheric greenhouse gases. Harvesting transfers considerable amounts of carbon to wood products, some of which can store carbon for extended periods (Chen et al. 2008). When carbon stored in harvested wood products between 2010 and 2100 was included in calculating carbon density for the HA harvesting scenario, carbon stocks decreased in only three of the 16 FMUs previously projected to have negative carbon density change (cf. Fig. 4), with revised carbon density changes for individual FMUs ranging from −10.7 t C ha^−1^ to 29.8 t C ha^−1^.

At the provincial scale (all 43 FMUs combined), the effect of forest age on projected carbon stocks could not be tested as the starting age structure was the same for all simulated scenarios. Hence, harvesting rate resulted in the maximum difference of 560 Mt C in forest carbon stocks in 2100 among the five simulation scenarios (Fig. 3A). However, the addition of carbon stored in harvested wood products (in use and landfill) narrowed this maximum difference to 89 Mt C, less than 2% of carbon stocks projected with the “lowest” scenario (HH). In year 2100, forest carbon stocks in the NH scenario are still noticeably higher than combined carbon stocks in forest and harvested wood product in the four scenarios including harvest. However, it may be argued that the difference is an artifact of the simulation time frame; forest carbon stocks in the NH scenario appear to be leveling off nearing the end of simulation period, thus making it likely that the total carbon stocks will further converge after 2100 (Fig. 3). This corroborates results by Colombo et al. (2012) who found for a subset of the management units in this study that combined carbon stocks in forest and harvested wood products effectively converged after 100 years of simulations with three scenarios involving “high”, “low”, and zero harvesting rate. Similar results were produced by Bradford (2011) who found carbon stored in wood products and landfill to partly compensate for the difference in forest carbon stocks among three scenarios including three levels of harvesting.

It is important to recognize that the comparison of nonharvested to harvested forest carbon and wood product stocks is strongly affected by the absence of natural disturbance in both scenarios. While removing nearly all disturbances from the forest by cessation of harvesting and suppression of fire maximizes forest carbon, it does so at the expense of ecological sustainability in these systems where large-scale disturbance is a natural event (Colombo et al. 2012). For this reason, our use of a protected forest comparative should not be considered a recommendation of this as a boreal forest management regime for climate change mitigation. In a comparison of a forest with a natural disturbance regime versus the same forest with reduced natural disturbance replaced by harvesting, combined forest plus wood product carbon stocks was higher in the harvested forest (Colombo et al. 2012).

It is also important to note that the convergence of harvested and nonharvested carbon stocks in forests and wood products applies specifically to the boreal forest. We do not anticipate a similar result in forests that consist of long-lived tree species in areas where natural disturbance rates are low. For example, in North American Pacific Northwest forests (Harmon et al. 1990) and tolerant hardwood forests of northeastern North America (Nunery and Keeton 2010) trees can live to greater age, continuing to store and sequester carbon with relatively low chance of natural disturbance compared to boreal forest species, and harvesting reduces combined forest plus wood product carbon stocks compared to forest that is not harvested.

Although combined forest product plus forest carbon stocks over time approached those had the forest not been harvested, considerable time was needed to approach carbon parity. Here and in Colombo et al. (2012), carbon stock parity required about 100 years. Carbon stock parity does not, however, take into account other factors affecting the parity of global warming potential caused by the combined effects of harvesting forests, including effects of transferring live carbon into dead forest carbon pools, life-cycle emissions, avoided emissions through product displacement, or changes in landscape albedo. While the atmospheric impact of traditional wood products has not received the same attention as has bioenergy from forest wood fiber (Helin et al. 2013), Pingoud et al. (2012) used the concept of “global warming potential factors” and “warming payback time” as indicators of the global warming impact of wood products. Such analyses are beyond the scope of this study, but optimizing climate change mitigation through forest management ultimately requires such a detailed approach to be taken.

Analyzed scenarios included harvesting rates ranged between zero and 2% per year of the available growing stock. These rates were dictated by environmental constraints (Ontario Ministry of Natural Resources [OMNR] 1996); higher harvesting rates may have a more pronounced effect on carbon stocks. However, for the range of considered values, we cautiously submit that harvesting rate in the boreal forest is not the most important factor for the long-term prediction of carbon stocks. Predicted changes in forest carbon stocks were poorly correlated with the harvest rate at the individual FMU level, whereas at the provincial level differences in forest carbon stocks were mostly compensated by carbon storage in harvested wood products in use and landfill.

The results of this study were based on several assumptions. First, analysis of projected carbon stocks did not account for emissions related to product life cycles, methane produced in landfills, and avoided emissions due to wood product displacement of more energy intensive materials. This shortcoming is to be addressed in our future studies on forest carbon storage. However, life-cycle emissions tend to be more than compensated for by-product displacement (Sathre and O'Connor 2010). While methane production from wood products disposed of in landfill can cancel out the mitigation value of such “stored” wood (Lippke et al. 2011), diverting waste wood from landfills and reusing it for other purposes would reduce this source of a potent greenhouse gas.

Second, harvesting scenarios were based on forest management plans that assumed volume of wood harvested using different methods remains constant throughout the simulation period. In 2011, three prevalent harvesting methods in Ontario (clear-cut, shelter wood, and selection) accounted for 85%, 8%, and 7% of total harvested area, respectively (National Forestry Database, http://nfdp.ccfm.org/silviculture/quick_facts_e.php, accessed July 2013). Although the effects of different harvest methods on carbon stocks has been studied at the stand level (e.g., Mund and Schulze 2006; Nunery and Keeton 2010), we have not found a landscape-level analysis of such effects on long-term dynamics of forest carbon stocks that includes carbon in harvested wood products. However, we anticipate that changes in harvesting methods or their use in proportions different from current practices may affect projections produced in this study.

Finally, the results of this study were based on predictions of future forest condition under the assumption that forest growth, successional change, and natural disturbance remain constant throughout the simulation period, which is a common approach used in large-scale timber supply models. Changes to the above rates caused by climate change or widespread natural disturbances would affect predicted forest condition and alter forest carbon stocks. This emphasizes the need to develop large-scale forest carbon models, either stand alone or coupled with timber supply models that account for the effects of changing climate and variations in natural disturbance regime.

In conclusion, the spatial differentiation of forest carbon sink or source activity across a large boreal landscape provides valuable information about nonuniformity of current forest conditions that strongly affect which forest areas can contribute to mitigating climate change. Some forest areas are more carbon dense, but as shown in Figure 4, this renders them ineffective to contribute further to carbon storage – in fact, the carbon dense areas are expected to be carbon sources between now and the end of the century. The most carbon-friendly management approach in such areas is probably a combination of protection from natural disturbances along with judicious harvesting to produce products with high displacement value. In areas that currently are relatively sparse in forest carbon due to previous human-caused or natural stand–replacing disturbance, a management approach with a significant component of forest protection will increase average forest age and carbon stocks. These approaches to mitigate climate change are not intended as management prescriptions. We have emphasized before that managing forests or harvesting for wood products to promote carbon stocks should be secondary to a philosophy of forest management that is centered on promoting ecological characteristics that foster sustainability at the stand and landscape levels (Colombo et al. 2012). Nevertheless, it is important to be aware that within the bounds of sustainable management choices are available that also contribute to mitigating climate change.
